# Enhancing Personalized Ads Using Interest Category Classification of SNS Users Based on Deep Neural Networks

**DOI:** 10.3390/s21010199

**Published:** 2020-12-30

**Authors:** Taekeun Hong, Jin-A Choi, Kiho Lim, Pankoo Kim

**Affiliations:** 1 Department of Computer Engineering, Chosun University, 309 Pilmun-daero, Dong-gu, Gwangju 61452, Korea; goodfax@chosun.kr; 2 Department of Communication, Department of Computer Science, William Paterson University of New Jersey, 300 Pompton Rd, Wayne, NJ 07470, USA; choij21@wpunj.edu

**Keywords:** SNS, personalized ads, deep learning, neural networks, interest classification

## Abstract

The classification and recommendation system for identifying social networking site (SNS) users’ interests plays a critical role in various industries, particularly advertising. Personalized advertisements help brands stand out from the clutter of online advertisements while enhancing relevance to consumers to generate favorable responses. Although most user interest classification studies have focused on textual data, the combined analysis of images and texts on user-generated posts can more precisely predict a consumer’s interests. Therefore, this research classifies SNS users’ interests by utilizing both texts and images. Consumers’ interests were defined using the Curlie directory, and various convolutional neural network (CNN)-based models and recurrent neural network (RNN)-based models were tested for our user interest classification system. In our hybrid neural network (NN) model, CNN-based classification models were used to classify images from users’ SNS postings while RNN-based classification models were used to classify textual data. The results of our extensive experiments show that the classification of users’ interests performed best when using texts and images together, at 96.55%, versus texts only, 41.38%, or images only, 93.1%. Our proposed system provides insights into personalized SNS advertising research and informs marketers on making (1) interest-based recommendations, (2) ranked-order recommendations, and (3) real-time recommendations.

## 1. Introduction

Among many variations and types of social media, social networking sites (SNS) are one of the most prominent internet-based applications that allow the exchange and creation of user-generated content. Social media have become deeply intertwined with consumerism. Social media provide platforms that consumers can use to search for information, interact with brands, share experiences, leave reviews, and even make purchases. Therefore, not only are almost all consumer brands present on social media, they actively engage in and integrate social media into their digital strategies [1]. Brands can establish themselves digitally through paid display advertising, creating brand personas, publishing branded content, and encouraging consumers to engage with their brands [2].

Consumers are exposed to thousands of advertisements daily [3]. Over time, consumers learn to avoid and reduce exposure to content and messages that do not interest them or are untrustworthy [4]. A way in which marketers can catch the attention of smart consumers is through personalized advertising. To help brands stand out to consumers in a digital environment inundated with an ever-growing clutter of advertisements, marketers use online consumer data to heighten the relevance of advertisements and to deliver them to targeted groups. Personalized advertising is a form of paid message that is “tailored to an individual’s characteristics and/or interests or tastes” [5]. Information on consumers is gathered and stored through web-based behavior-tracking and database technology, such as tracking users’ web browsing behavior, use of cookies, implementation of opt-in and opt-out options on websites, and requiring an agreement to personal data collection as a condition to use SNS platforms [6]. Marketers then use consumers’ information to strategically devise advertisements.

The ability to garner consumer data online is crucial as personalized communication is known to be an effective persuasion strategy that positively influences attention, cognitive responses and attitudes toward advertisements [7]. Research shows that personalization is known to generate favorable responses as consumers prefer advertisements that are relevant to them, whereas SNS advertisements that are not relevant to the user are likely avoided as they are perceived as disruptive or invasive [4,5]. Therefore, previous research supports the need for personalization in the digital environment as it improves advertising effectiveness, such as being more memorable and more likeable, increasing motivation to process advertisements, initiating behavioral change, and improving advertising response rate [8,9].

One of the advantages of advertising on SNS is the ability to send advertisements to specific target groups based on self-disclosed information found on public profiles, such as age and gender. However, there is limited research on personalized SNS advertisements, and the research needs to go beyond demographics and explore information unique to social media [1]. Currently existing methods for extracting consumer information may give marketers limited or even inaccurate information about consumers. As SNS profiles are built on voluntary self-disclosure of personal information, there are opportunities for intentional misrepresentation which opens up avenues for fake profiles, internet trolls and bots. Therefore, SNS data aggregation can lead to inaccuracies in consumer profiling and targeted communication. Personalization based on such data subsequently leads to a decrease in the marketer’s ability to accurately predict relevance of advertisements to consumers. Additionally, mainly textual features of user-generated content on SNS, largely Twitter, have been used in research on personalized and target advertising [10]. There is a dearth of research using images on social media as well as research on classifying the interests of SNS users by analyzing images.

Therefore, this research bridges the gap in current research on personalized advertising. In addition to gathering information regarding an individual’s characteristics, the ability to gauge a consumer’s actual interests would unquestionably enhance personalized advertising. Although the adverse effects of irrelevant advertisements have been researched extensively [4,5,11], research on personalized advertising using SNS consumer information extending beyond demographics and online user behavior, such as web-browsing cache, has yet to be explored. This is perhaps due to the fact that gauging the constantly evolving interests of millions of SNS users is a seemingly daunting and unachievable task. Therefore, the current research aims to bridge the gap in personalized advertising research by proposing a machine learning-based method to identify SNS users’ interests in order to, by the definition of personalized advertising, tailor it to users’ “interests or tastes” [5]. As previous research shows, increasing perceived relevance results in a more positive consumer response [7,9].

Furthermore, previous studies examining SNS data have focused largely on textual analysis and less on using images. However, SNS platforms today rarely host either texts or images alone, but a combination of both. Users post images and texts together intentionally, assigning related and specific meanings to constitute a single post. Therefore, when analyzing SNS data, it is best to consider both text and image-based data together to accurately determine the intentions behind the users’ SNS posts. Additionally, to enhance previous studies on SNS users’ interest classification, this paper analyzes SNS platforms where images and text are posted together to perform classification of users’ interests to be applied to personalization advertising.

The current paper proposes the analysis of user-generated data on SNS, particularly user-generated posts with both images and texts, using deep-learning to identify and classify SNS users’ interests into suggested categories for advertisers.

Using a convolutional neural network (CNN)-based model for image classification, and a recurrent neural network (RNN)-based model for text classification, models with the highest accuracy were used to create a hybrid neural network (NN) model in order to classify user interests based on results of training datasets. Finally, classification accuracies were measured for text only, image only, and text–image combination for classification of SNS user interest. This paper aims to offer marketers insights into personalized advertising by rank ordering SNS users’ most recent interests in real-time.

The remainder of the paper is organized as follows: Section 2 provides background of our work, and Section 3 presents our proposed user interest classification system based on deep neural networks. Section 4 discuss the experimental methods and experiment results, and Section Section 5 discuss the significance and implication of our work. Finally, we conclude in Section 6.

## 2. Related Works

### 2.1. Previous Works on Personalized Advertising

Targeting of consumers with personalized messages is a fundamental strategy in advertising practice that has been researched extensively since the emergence of mass media [12,13,14]. Personalization of advertisements has since evolved to incorporate machine learning-based approaches.

Today, new artificial intelligence technologies and recommender systems are used to identify potential consumers [6]. Using the content-based approach, Mooney and Roy [15] examines the degree of similarity between consumers and product content profiles, examining parsable information, such as textual data. Mining textual features of user-generated content on SNS, largely Twitter, have been frequently used to predict and target audiences [10], as well as the discovery of blogger’s personal interests [16] and content-based recommender system for SNS users with relevant interests and preferences [17]. Keyword extraction from online content can find meaningful language patterns [18], and when consumer information is not available, audience attributes such as age, gender and personality are detected with machine learning classifiers [19].

A data-mining framework using social networks to construct a targeted advertising system uses social interaction data [20]. Furthermore, collection of behavioral data, such as likes, comments, searches and tweets, helps to make inferences on consumer preferences [21]. Collaborative filtering is used to compare profiles of users in predicting possible interests of users based on similarity of preferences of ratings on items [22], while similarity measurement methods, such as the Pearson correlation coefficient, are also used to identify users based on ratings [23]. Furthermore, predicting users’ preferences can be identified using graph-based approaches and purchase time information [24].

As exemplified above, much previous research on personalized and targeted advertising has focused on text mining. However, the SNS scenery is becoming more dynamic with image-based user-generated content. With popular applications, such as Instagram, which are primarily based on images versus textual data, there is a pressing need to gauge consumers’ interests using user-generated content focused on pictures instead of relying solely on text mining and keyword extraction. Image recognition is known to help advertisers comprehend true consumer behavior through the examination of pictures [21]. In addition to posting pictures, SNS users also self-identify descriptions for the picture, providing textual and contextual details which enhance the images [21]. Pictures can describe a thousand words, such that even when not explicitly stated, image recognition can identify and reveal brands and products [21]. Therefore, the present study examines both images and texts from SNS users to enhance precision of identifying consumers’ interests.

### 2.2. Previous Works on Interest Classification

Classifying user interests is one of the most important steps in personalized advertising as it provides information about users’ interests that could be used by marketers or advertisers. There have been various studies to classify users’ interests [25,26,27,28,29]. A study on SNS suggests a classification method to classify users’ active communication through comments into a grading system using Word2vec and support vector machine (SVM) classifier [25]. The weighting ensemble model was proposed to classify the user’s emotions into multi-label binaries by analyzing the content created by the user, and this model performs the classification result without hyperparameter adjustment or overfitting [26]. Consumers buy products online, and there are many user comments regarding their decision of whether or not to make a purchase. However, users’ comments are categorized using topic modeling and classification techniques to address the inconvenience that users face in reading numerous comments daily [27]. Another study uses deep learning to predict the probability of clicking an ad by associating it with user interests to predict the user’s interest and response to ads shown online [28]. In order to provide convenience to email users, e-mails are classified through the segmentation of intent in e-mail. With this, a way to identify spam e-mails that commit malicious acts is suggested [29].

However, there is very little research using images on social media as well as research on classifying the interests of SNS users by analyzing images. An image-based study classified user’s emotions and suggested that most of the existing emotional classification studies consider only the position of the viewer and that the amount of image-based data is small. Taking this into account, the post’s image and hashtags were used to classify emotions of the SNS user [30]. Another study analyzed images posted on SNS and classified the SNS platforms to which the images were posted, as the impact on cybersecurity is significant according to SNS platforms [31].

As mentioned above, most studies related to classifying user interests on SNS are conducted using text. There are studies using images on SNS, but most of them are not related to users’ interests. However, both text and images should be used to determine the intention behind users’ SNS post. Therefore, it is important to use text and images together to classify users’ interests. This is important because using texts and images together to classify users’ interests can more accurately determine their intentions and thoughts. Therefore, in this study, we propose to classify user interests on SNS by utilizing both texts and images of user-generated contents posted by users.

### 2.3. CNN and RNN-Based Classification Models

This section describes the models used to classify interests using images and texts extracted from user posts. In the proposed hybrid model, images and text are grouped separately, and then CNN-based image classification models and RNN-based text classification models are used. The CNN models used for categorizing users’ interests through images include Inception res v2 [32], MobileNet v2 [33], Inception v3 [34], ResNet v2 [35], and EfficientNet b7 [36]. RNN models used for categorizing users’ interest through texts include basic recurrent neural networks (RNNs) [37], long short-term memory (LSTM) [38], the gated recurrent unit (GRU) [39], and bidirectional LSTM [40].

#### 2.3.1. Models Based on CNN

The Inception res v3 [32] model combines the traditional image classification model, ResNet, and Google Incidence. Studies have been conducted to combine models in various ways to improve image classification performance. However, with the combination of models, the size of the model has grown and, thus, does not perform well when compared to the larger computations and learning times. In the Inception res v3 models, to improve these problems, a simplified input model is proposed and combined with ResNet, which shows excellent performance. MobileNet v3 [33] is a deep learning model that performs well in image classification in limited environments such as mobile devices. To do this, there is a need to significantly reduce the computations and depth of models compared to conventional models. In MobileNet v1, a model suitable for significantly reducing computations and model depth was proposed by introducing the concept of deep-flow separable convolution. MobileNet v2 has an inverted residual structure as a way to change the model structure to improve the performance of MobileNet v1, which reduces the amount of computation and improves performance. Inception v3 [34] has the same structure as the Inception v2 model announced by Google but has been indicated to be the most capable model through several learning methods. This was proposed to compensate for the shortcomings that were not widely used due to the need for a large number of parameters, despite the small number of parameters compared to VGGG and AlexNet, which were released together at the time of the publication of Inception v3.

While most of the models proposed for image classification have very deep layers and perform well in many operations, ResNet [35] offers a residual layer. This method reduces computations and improves performance by skipping layers when performing operations, even for models with deep layers. In deep layer models, ResNet v2 provides methods to reduce computations and improve performance by applying the bottleneck structure with 1D convolution. The EfficientNet [36] model was proposed amid a period of little research interest regarding image classification and lack of research beyond traditional performance. With an emphasis on model scaling, the developers of this model suggested adjusting its size through width scaling, depth scaling, and resolution scaling. Width scaling is a method of controlling the number of layers; depth scaling is a method of controlling the resolution of an input image. The previously proposed image classification model, ResNet, is a method of regulating depth scaling, and MobileNet is a representative method of regulating the model through width scaling, but it is rare to consider width, depth, and resolution scaling at the same time as in the case of EfficientNet, which appears to be the best-performing model.

#### 2.3.2. Models Based on RNN

The RNN-based model [37] is a deep learning model used in time-series data that change over time, such as natural language, and it aims to continuously store information on inputs that vary over time and produce the most appropriate outputs that can appear at this point in time to the next. LSTM [38] is a proposed method to address long-term dependency problems arising from RNN. Because RNN has a simple structure that continuously repeats information from past and present times in the same way, the problem of losing information from a very long past exists. To improve this, a gate was added to LSTM to selectively flow information from the previous point in time to anticipate or learn the next point at this point. In this way, LSTM consist of cell state, forget gate, and input gate. GRU [39] is a model that has the same purpose as the proposed LSTM to address the long-term dependency problem, which is the problem of losing old-time information. GRU provides an update gate and reset gate, and although its performance is similar to LSTM, the fast learning speed is an advantage. While LSTM adjusts the amount of information to be handed over to the next layer through the forget gate, GRU improves the calculation speed by choosing to either hand over the current information to the next layer or not at all through the reset gate.

While RNN, LSTM, and GRU are models for learning only from the past and predicting the future through current-time information, bidirectional LSTM [40] represents a model that adds a backward state layer for learning future information to improve future predictive performance. For example, when the sentence “I am a boy” is present, existing RNN-based models learn “I am a boy” the same as forwarding state layers and, in addition, “boy a am I” in the backward state layer of bidirectional LSTM. This will help preserve past and future information at the same time and better predict future information.

## 3. Interest Category Classification of SNS Users Based on Hybrid NN Models

This section describes how to classify the interests of SNS users. Curlie [41], an Open Directory Project (ODP), was re-defined and used as a category of interest as a criterion for classifying users’ interests. The proposed method consists of two main parts. The first part is the training model for classifying user interest. It collects images and texts as keywords for categories of interest on SNS posts and web pages.

The next step is to learn CNN-based models for image classification and RNN-based models for text classification. The CNN-based models that were used include Inception res v2, MobileNet v2, Inception v3, ResNet v2, and EfficientNet b7, and the best performance model based on accuracy was selected. The RNN-based models that were used for text classification include RNN, LSTM, GRU and bidirectional LSTM, and, as with image classification, the best performance model based on accuracy was selected. These selected CNN model and RNN model were used as a hybrid NN model for user interest classification next.

**Figure 1:**
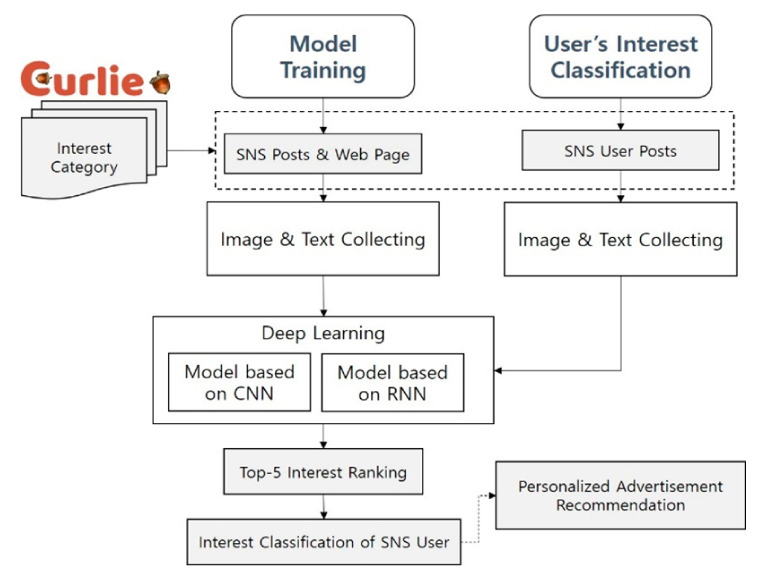
Architecture of category classification system for personalized ads.

The second part is the user interest classification with the learned image and text model. This collects images and texts from SNS users whose interests fall in the categories of interest. This part takes the user’s images and texts as inputs into the model learned in the model training process. At this point, five results are obtained through top-5 interest ranking, and these are classified through images and texts. Since it is likely that each user has more than one interest based on their posts, the proposed method classifies five interests for each user. This allows our proposed system to be used for personalized advertisement and recommendation. The system architecture of the interest category classification of SNS users based on hybrid NN models proposed in this paper is shown in Figure 1.

### 3.1. Definition of Interest Category

In this paper, Curlie [41] is utilized as a criterion when classifying users’ interests. Curlie started with an ODP called DMOZ that Internet users voluntarily participate in. Based on hierarchical ontology, it lists sites by category. Curlie, similar to the directory structure, is organized into categories of top-to-bottom relationships, and it has semantic features because users voluntarily participate. Thus, based on these features, this paper selects categories suitable for the classification of user interests and defines them as categories of interest. The categories of user interests are shown in Table 1.

### 3.2. Training Dataset

A training dataset is used to train CNN-based image classification models and RNN-based text classification models for the classification of interests of SNS users. Training data consist of images and text. The dataset collects results of searching interest categories as keywords on SNS platforms or portal sites on the Web, such as Instagram, Twitter, Facebook, Flickr, and Google.

### 3.3. CNN and RNN Classification Models

In this paper, CNN-based models were used for images and RNN-based models were used for texts to classify the interests of SNS users. Several CNN-based models and several RNN-based models were compared and analyzed in terms of performance and accuracy, and then the best performed models were selected for a hybrid NN model to classify user interests based on images and texts of SNS users.

#### 3.3.1. Models Based on CNN

This section describes the structure of the model for classifying users’ interests through images. The CNN-based image classification models used in image classification include Inception res v2 [8], MobileNet v2 [9], Inception v3 [10], ResNet v2 [11], and EfficientNet b7 [12]. Using the pre-trained model of each model, transfer leading was performed on the image dataset corresponding to the category used. We selected models that show excellent performance in image classification, and the learning results of these models use one model that is superior to the classification of user interests.

**Figure 2:**
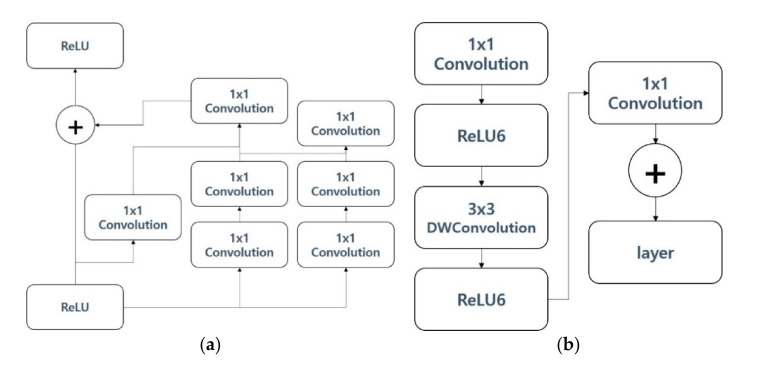
(**a**) Inception res v3 model; (**b**) MobileNet v2 model.

The Inception res v3 model [8] combines the traditional image classification model, ResNet, and Google Incidence. In the Inception res v3 model, a simplified Inception model was proposed to improve the problem of many computations occurring at the depth of the deepening model, and combined with ResNet, it showed excellent performance. Figure 2a shows the structure of the pre-trained Inception res v3 model used in this paper. The solution blocks on the right are from the Inception v3 model and the left-hand Rectified Linear Unit (ReLU) and its operations are from ResNet. MobileNet v2 is a deep learning model that performs well in image classification in limited environments such as mobile devices. MobileNet v1 focuses on significantly reducing computations and model depth. MobileNet v2 has an inverted residual structure as a way to change the model structure in order to improve the performance of MobileNet v1, which reduces the amount of operation and improves performance [9]. Figure 2b displays the structure of the pre-trained MobileNet v2 model used in this paper. MobileNet v2 has many similarities to the structure of ResNet in that it uses ReLU as an activation function in the middle of the filter and add operations. DWConvolution is an abbreviation for depth-wise convolution, in which each filter is fixed by the formula of width*height*depth = 1, and convolution is performed independently for each image entered.

**Figure 3:**
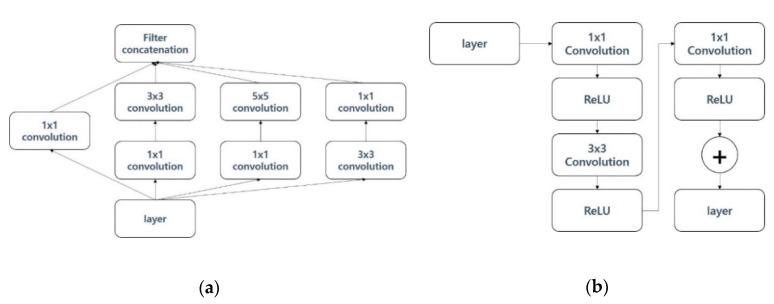
(**a**) Inception v3 model; (**b**) ResNet v2 model.

Inception v3 was proposed to compensate for the shortcomings regarding the problem of requiring a large number of computations, although the number of parameters to be computed was small compared to VGGG and AlexNet, which were released together at the time that Inception v2 was published [10]. Figure 3a shows the structure of the pre-trained Inception v3 model used in this paper. The Inception v3 model consists of a combination operation and a connection between layer and layer. The size of the filter consists of various solutions ranging from 1 to 5. The image classification model has deep neural networks. ResNet has a residual layer that has deep neural networks, allowing it to improve classification performance while reducing the amount of operations [11]. Figure 3b displays the structure of the pre-trained ResNet v2 model used in this paper.

The ResNet v2 model not only reduced computations but also improved performance by skipping layers during operations, despite the fact that it is a deep layer model. ResNet v2 provides a way to reduce the amount of computation and improve performance by applying a bottleneck structure with a 1D convolution in a deep layer model.

**Figure 4:**
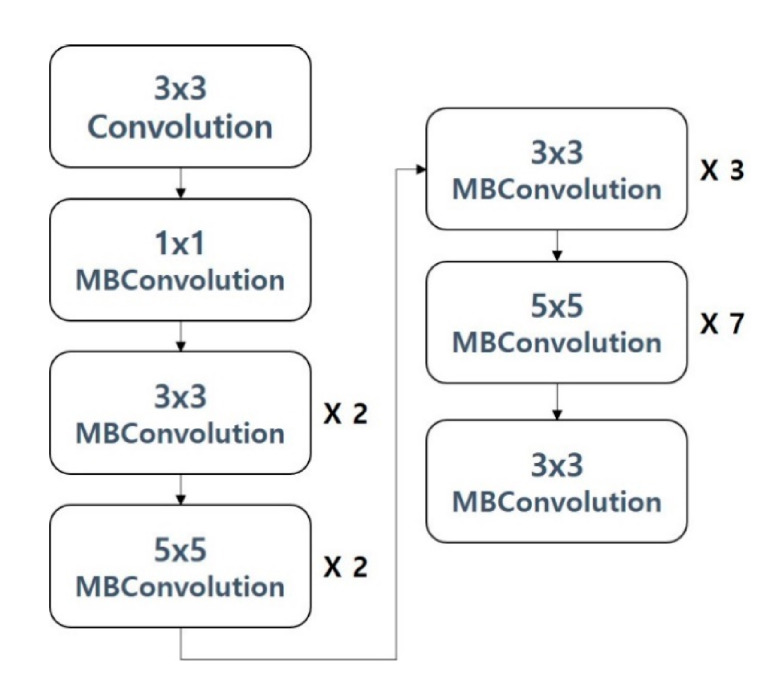
EfficientNet b7 model.

EfficientNet [12] was proposed to adjust the size of the model through width scaling, depth scaling, and resolution scaling, with a focus on modelling. Width scaling is a method of controlling the number of layers, while depth scaling is a method of controlling the resolution of the input image. Previously, among the proposed image classification models, there were models that controlled depth scaling and width scaling, but few models considered width, depth and resolution scaling at the same time. Figure 4 shows the structure of the EfficientNet b7 model used in this paper.

In this paper, transfer leading was carried out using the pre-trained model of the previously described Inception res v2 [32], MobileNet v2 [33], Inception v3 [34], ReNet v2 [35], and EfficientNet b7 [36]. Through the accuracy and loss derived through this, the most suitable models were selected and utilized in the classification of user interest.

#### 3.3.2. Models Based on RNN

In our hybrid NN model, RNN models used for classifying users’ interests through text include basic recurrent neural networks (RNNs) [37], long short-term memory (LSTM) [38], the gated recurrent unit (GRU) [39], and bidirectional LSTM [40]. The CNN-based models used for image classification transfer learning image data suitable for user interest classification in pre-trained models, but the RNN-based models used for text classification implement a basic and simple structure and were used for the user interest classification. Then, the model with the best learning outcomes was selected.

**Figure 5:**
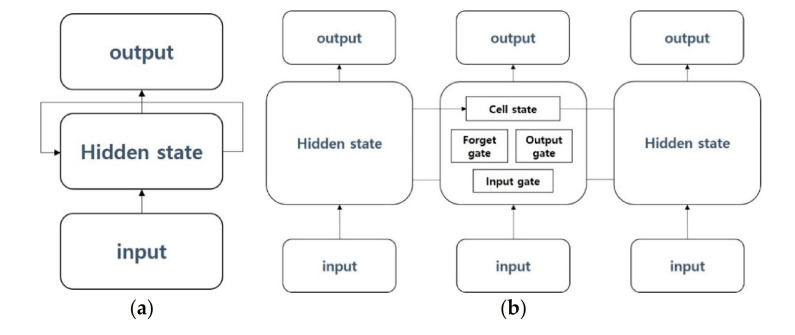
(**a**) Vanilla recurrent neural network (RNN) model; (**b**) long short-term memory (LSTM) model.

One of the models considered is the most basic vanilla RNN, called simply the RNN [37] model. At the experimental stage, the model was built by only setting the number of layers and the dimension of input. Figure 5a shows the structure of the used vanilla RNN model.

**Figure 6:**
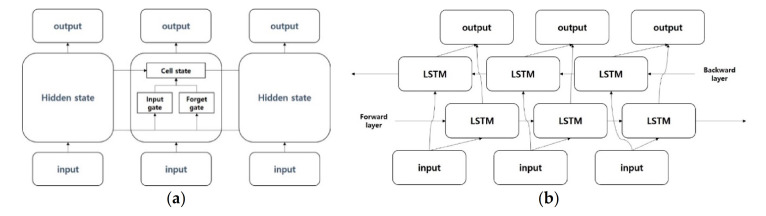
(**a**) Gated recurrent unit (GRU) model; (**b**) bidirectional LSTM model.

The second model considered in the classification of text interest is LSTM [38]. LSTM is a proposed method to address long-term dependency problems arising from RNN. LSTM offers cell state, forget gate and input gate, which solve the long-term dependency problem. Figure 5b displays the structure of the LSTM model used in this paper. The GRU [39] is a model that has the same purpose as the proposed LSTM to address the long-term dependency problem, which is the problem of losing old-time information. GRU has update gate and reset gate, which are similar in performance to LSTM but have advantages in fast learning. Figure 6a displays the structure of the GRU model used in this paper.

While RNN, LSTM and GRU are models for learning only from the past and predicting the future through current-time information, bidirectional LSTM [40] proposes a model that adds a backward state layer for learning future information to improve future predictive performance. This will help preserve past and future information at the same time and better predict future information. Figure 6b shows the structure of the bidirectional LSTM model used in this paper.

### 3.4. User Dataset

A user dataset is used to classify user interests. It is an input to our hybrid NN model learned in previous steps. The training datasets consist of images and text. The user datasets, however, are collected from user-generated content on the SNS platforms by selecting any user with sufficient images and text to classify their interests. Note that only users with more than 100 posts were included in the user dataset in this paper.

### 3.5. Method of Classifying SNS Users’ Interests

This section describes the interest classification of SNS users in detail. Image and text interests are categorized, respectively, by inputting posts from SNS users in the CNN-based image classification model and the RNN-based text classification model described in Section 3.2. At this point, SNS user’s accounts with sufficient number of posts (e.g., at least 20 posts) are required to carry out the classification of interests. In order to identify the most accurate classification of users’ interests, texts-only, images-only or the combination of texts and images together were examined. From the results, the top-5 interests in each case were ranked to classify five interests. This was carried out because people’s interests are not consistent with one thing, and users’ interests may vary. In addition, in this paper, we evaluated the performance of the classification accuracy rate of user interest for the case where only text was used in the classification of user interest, where only image was used, and where text–image combination was used by a comparative experiment to verify the best-performing method.

### 3.6. Method of Personalized Advertisement Recommendation

The aforementioned methods can be used to classify the interests of SNS users. In order to classify the interests of SNS users, the interests were classified through a hybrid NN model. In addition, the proposed approach can help marketers who provide personalized advertisement recommendations for SNS users by using classified SNS user interests.

## 4. Experiment and Analysis

This section assesses the performance of CNN-based image classification models and RNN-based text classification models conducted for classifying categories of user interest through accuracy and loss. Through this assessment, we select the image classification and text classification model that best suits the user interest classification categories proposed in this paper. The performance evaluation of the selected model is used to classify user interests using texts only, images only, and text–image combination to create a top-5 interest ranking. Tensorflow 2.3.1 with Python 3.8.5 was used in this study as the deep learning platform.

### 4.1. Image and Text Collection

The image collection and text collection were divided into two main categories: data for use in training deep learning models and data on SNS users to classify interests.

Training data used for training and user data used for classifying user interests are completely independent parts. Images for training images of deep learning models were collected from Instagram, an image and text-based SNS platform; Flickr, an image-based SNS platform; and Google, a portal site that provides image search results. Text for training text from deep learning models was collected on Twitter, an SNS platform that uses short text. The images and text used for training were collected from a search based on the interest category. The training dataset consisted of 33,647 images and 21,022 pieces of text. The text was then organized into sentence units.

Users data, which were used to classify user interests, targeted Instagram users. Users with more than 100 postings were randomly selected based on the predefined interest category from Curlie. Then, the 20 most recent posts were used to classify the Instagram users’ interests. In this paper, we classified the interests of 29 users. The user dataset consisted of 580 images and 1329 pieces of text. Like the training dataset, the text was organized into sentence units. Note that each post consisted of one image and one content (text), and the text classification was performed on a sentence basis.

### 4.2. Performance Comparison of Models Based on CNN

In this section, we examine the CNN-based image classification models used in this paper and select the model with the best image classification performance results. The models for performance evaluation are EfficientNet b7, ResNet v2, Inception v3, MobileNet v2, and Inception res v2. Table 2 displays the hyperparameter used to examine the CNN-based models.

**Figure 7:**
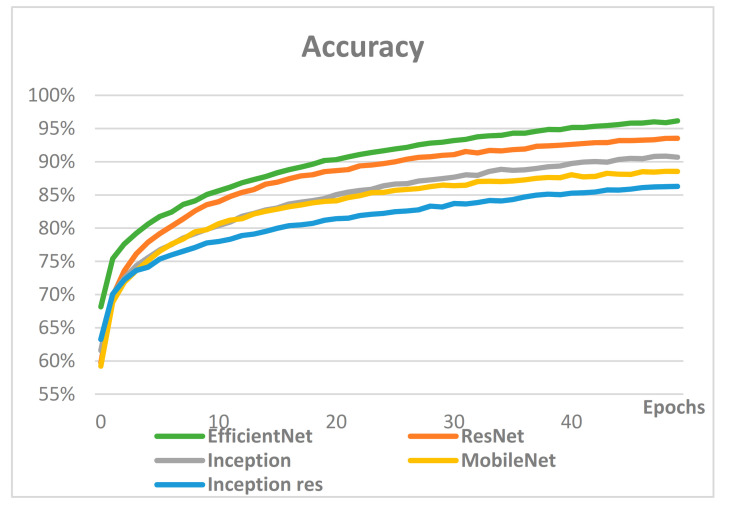
Accuracy comparison of the CNN-based models.

**Figure 8:**
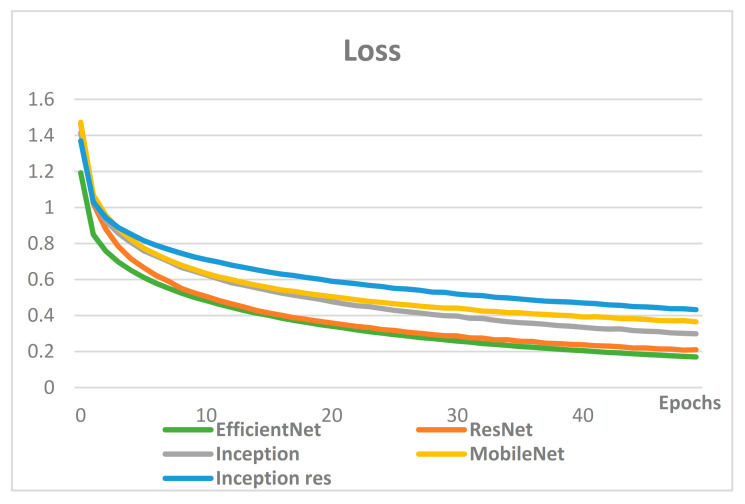
Loss comparison of the CNN-based models.

Most models were studied with similar hyperparameters, and performance comparisons were conducted mainly on models known to have excellent performance in image classification. Figure 7 compares the accuracy of CNN-based models, while Figure 8 compares loss.

The comparison of accuracy and loss in CNN-based models shows that the two indicators are proportional, and the best performing model is EfficientNet. The accuracy of EfficientNet, ResNet, MobileNet, Inception and Inception res was 96%, 94%, 91%, 89% and 86%, respectively. Therefore, EfficientNet was selected for the proposed classification of user interests for image classification.

### 4.3. Performance Comparison of Models Based on RNN

We examined RNN-based text classification models and selected the model with the best text classification performance through performance results. The models for performance evaluation are bidirectional LSTM, GRU, LSTM and RNN. Table 3 shows the hyperparameter used to examine the RNN-based models.

**Figure 9:**
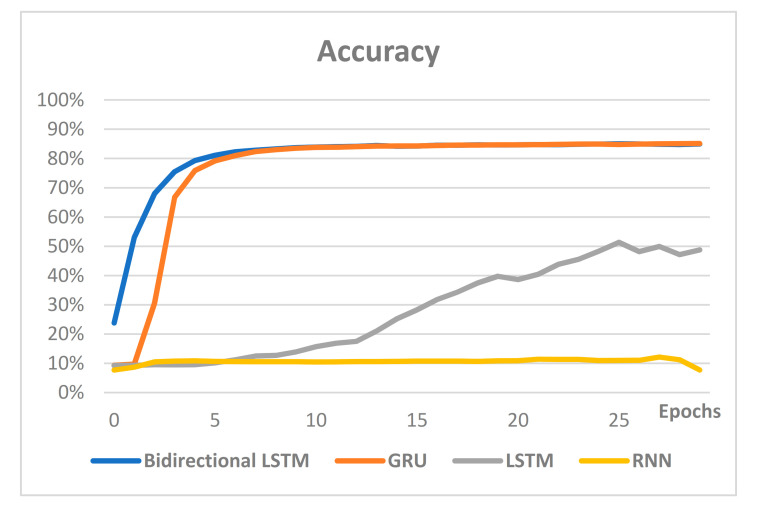
Accuracy comparison of the RNN-based models.

**Figure 10:**
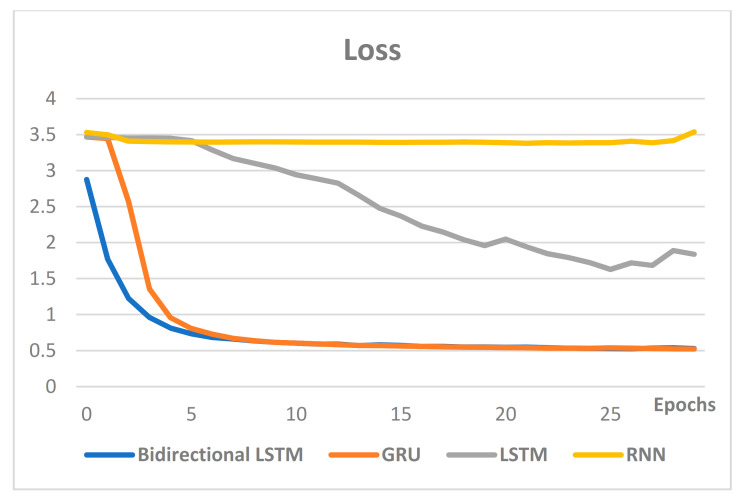
Loss comparison of the RNN-based models.

Most of the models were studied with similar hyperparameters, and the performance comparisons were conducted on the models that were mainly used for text. Figure 9 compares the accuracy of the RNN-based models, while Figure 10 compares loss.

Comparison of the accuracy and loss of the RNN-based models shows that the two indicators are proportional, and the model showing the best performance is bidirectional LSTM. The accuracy of bidirectional LSTM, GRU, LSTM and RNN was 86%, 85%, 49% and 8%, respectively. Therefore, bidirectional LSTM was utilized for the classification of user interests for text classification in this paper.

### 4.4. Classification of SNS Users’ Interests

Finally, the classification of user interests was measured in three instances: when only texts were used to categorize users’ interests, when only images were used, and when texts–images were used together. This process verifies which datasets have the best performance. Baseball, cooking, fitness, golf, pets, renovation, and vehicles were selected as the test sets in the entire category to carry out the classification of interests, with a total of 29 users selected. Table 4 shows an example of performing a classification examination for golf.

Table 4 shows classification of interests based on the text, image, and text–image combination in SNS users’ posts that are labeled as interested in golf.

As described earlier, five interests were classified and rank-ordered for each dataset through top-5 ranking. In Table 4, for user 1, because golf is revealed as an interest category for text, image, and text–image combination, all three methods accurately classified the user’s interest.

We measured the accuracy of the classifications of user interest for a total of 29 users using text, image and text–image combination. The results are as shown in Table 5.

The classification of user interest with text, image, and text–image combination and measurement of accuracy were performed for a total of 29 SNS users. The use of text–image combination showed the best results, with text showing very poor results at only about 41%. This seems to be due to the nature of SNS. Users often write hashtags or text messages that are not necessarily descriptive of the images or the contents of their post. On the other hand, images alone seem to have excellent performance because of the clear content and purpose. Therefore, regardless of how good the classification performance of interest using text is, there is clearly a small number of useful pieces of information contained within the textual data. Therefore, rather than using texts or images separately, it seems that using texts and images together will produce better performance.

## 5. Significance and Implication

The present research offers several new implications and insights into research on personalized advertising on SNS with the proposed method, providing marketers with (1) interest-based recommendations, (2) ranked-order recommendations, and (3) real-time recommendations. The proposed method takes a different approach to personalized advertisements on SNS which heavily rely on users’ information, such as self-disclosed information from their public profiles such as demographics, and online behaviors as exemplified by web-browsing history. With the current method, marketers can tailor advertisements based on actual interests and behaviors displayed by the consumer. It also provides marketers with ranked-order suggestions for categories that SNS consumers are interested in for a wider range of brand recommendations. Using the hybrid NN model-based classification system discussed above, marketers can determine consumers’ interests in real-time with heightened accuracy.

Although recent research examines user-generated content, much focus has been on textual data on SNS. SNS posts, however, provide rich information through both images and text. Therefore, the proposed method analyzes images and texts together, allowing reduction in errors caused by text-only-based approaches in personalized advertising on SNS. To the best of our knowledge, this article is among the first to use both image and text-based data from user-generated content to improve the ability to accurately identify actual interests of SNS users for the purpose of enhancing the experience of personalized advertising.

The large media fragmentation in today’s digital environment increases the potential to target certain consumers with personalized messages. Scholars and professionals alike acknowledge the vital importance of personalization for success in business. Therefore, there is an imperative need for examination of methods that enhance the accuracy of personalization. The ranked-order identification and classification of individuals’ distinctive interests in real-time allow the marketer to make an informed decision based on the consumer’s most recent set of interests.

Furthermore, we compare and evaluate the performance of various classification models to find the best suited image classification model and text classification model for the hybrid NN model to address the research problem. Classification models can be developed with different input data depending on the target goals. Even with a classification model with proven performance, the performance of the model could differ depending on the input data. Therefore, we measured benchmarks of multiple classification models to select the best model for the proposed method in this paper.

## 6. Conclusions

With growing numbers of SNS users who create content, search for information, share experiences, leave reviews, and purchase items through SNS, it is critical to enhance personalization of advertising messages by precisely identifying and classifying consumers’ interests to avoid phenomena such as ad avoidance. Most existing research on classification of interests uses only textual information from SNS user-generated posts, such as the users’ comments or image descriptors, while few use images. However, the combination of texts and images on user-generated posts is predicted to optimize the performance of the classification of consumers’ interests on SNS, as it utilizes all of the information provided by the user’s SNS page. Thus, in this paper, we propose an interest classification system using texts and images of SNS users’ postings together. Interests were redefined using Curlie. To classify users’ images, a CNN-based classification model was used, and an RNN-based classification model was used for textual data. CNN-based classification models were selected as candidates, namely, Inception res v2, MobileNet v2, Inception v3, ResNet v2, EfficientNet b7, and they resulted in accuracies of 86%, 89%, 91%, 94%, and 96%, respectively. Therefore, the EfficientNet b7 model was used as the image classification model based on CNN for user interest classification. RNN-based classification models that were selected as candidates were RNN, LSTM, GRU and bidirectional LSTM. As a result of learning, accuracy of each model was 8%, 49%, 85% and 86% in order. Therefore, the RNN-based text classification model for classifying user interests used bidirectional LSTM. Finally, text-only was used to categorize users’ interests with 41.38% accuracy. The classification of user interests was measured with 93.1% accuracy when only images were used, and 96.55% accuracy when texts and images were used together. The results of the experiments showed that utilizing all information on the user’s SNS post, including texts and images together, best reflects the user’s interest.

## Figures and Tables

**Table 1:** Categories of user interests.

Dance	AI	Comics	Smartphone	Nutrition	Camping
Clothes	Hair	Gardening	Fishing	Pets	Hunting
Vehicle	Baseball	Art	Golf	Book	Laptop
Basketball	Skincare	Spa	Renovation	Cooking	Furniture
Ski	Travel	Disability	Football	Fitness	Electronics
Movie	Tattoo	Food	Swim	Music	Jewelry

**Table 2:** Hyperparameter used to examine the models based on CNN.

	Efficient Net-b7	Res Net v2	Inception v3	Mobile Net v2	Inception Res v2
Image shape	600,600	224,224	299,299	224,224	299,299
Classifier	Softmax	Softmax	Softmax	Sortmax	Softmax
Optimizer	Adam	Adam	Adam	Adam	Adam
Loss function	Crossentropy	Crossentropy	Crossentropy	Crossentropy	Crossentropy
Activation function	SELU	ReLu	ReLu	ReLU6	ReLu
Pretrain dataset	ILSVRC-2012-CLS	ILSVRC-2012-CLS	ILSVRC-2012-CLS	ILSVRC-2012-CLS	ILSVRC-2012-CLS
Drop out	0.5	0.5	0.5	0.5	0.8
Epochs	50	50	50	50	50

**Table 3:** Hyperparameters used to examine the models based on RNN.

	Bidirectional LSTM	GRU	LSTM	RNN
Input dimension	64	128	128	128
Classifier	Sortmax	Sortmax	Sortmax	Sortmax
Optimizer	Adam	Adam	Adam	Adam
Loss function	Crossentropy	Crossentropy	Crossentropy	Crossentropy
Activation function	ReLu	ReLu	ReLu	ReLu
Vocabulary size	10,000	10,000	10,000	10,000
Epochs	30	30	30	30

**Table 4:** Examples of classifying users’ interests.

Label: Golf		Classification Result		
**User 1**	**Rank**	**Text**	**Image**	**Text–Image**
	1	furniture	***golf***	***golf***
	2	movie	design	design
	3	***golf***	ai	furniture
	4	clothes	baseball	movie
	5	comics	basketball	book
**User 2**	**Rank**	**Text**	**Image**	**Text–Image**
	1	hair	***golf***	***golf***
	2	laptop	music	music
	3	cooking	tattoo	hair
	4	diet	dance	laptop
	5	disability	smartphone	cooking
**User 3**	**Rank**	**Text**	**Image**	**Text–Image**
	1	pet	movie	movie
	2	clothes	***golf***	***golf***
	3	comics	music	music
	4	fishing	dance	dance
	5	book	music	cooking

**Table 5:** Results of classification of all users’ interests.

	Text	Image	Text–Image
**Accuracy**	41.38%	93.10%	96.55%
